# Supplementary material for: Performance Improvement and Biofouling Mitigation in Osmotic Microbial Fuel Cells via In Situ Formation of Silver Nanoparticles on Forward Osmosis Membrane
Source: Membranes (Basel). 2020 Jun 16;10(6):122. doi: 10.3390/membranes10060122 (PMC7344936; doi:10.3390/membranes10060122)
Supplement: Supplementary file 1 [file membranes-10-00122-s001.pdf]

Cover Page for Supplementary Materials

Manuscript title:

Performance improvement and biofouling mitigation in osmotic microbial fuel cells via *in situ* formation of silver nanoparticles on forward osmosis membrane

Author list:

Yuqin Lu[1], Jia Jia[2], Hengfeng Miao[1], Wenquan Ruan[1], Xinhua Wang[1,*]

[1] Jiangsu Key Laboratory of Anaerobic Biotechnology, School of Environmental and Civil Engineering, Jiangnan University, Wuxi 214122, P.R. China

[2] Zhejiang Province Environmental Engineering Technology Appraisal Center, Hangzhou 310012, P.R. China

*Corresponding author. E-mail: xhwang@jiangnan.edu.cn (X. Wang); Tel: +86-510-85326516.

Total number of pages (including the cover page): 11 pages

List of Tables and Figures:

Page 3 **Figure S1 Schematic diagram of the OsMFC.**

Page 4 **Table S1 Composition of the synthetic wastewater.**

Page 5 **Table S2 EDX analyses of the pristine and modified active layer of TFC-FO membranes.**

Page 6 **Figure S2 AFM micrographs (a) and contact angle micrographs (b) of the pristine (1) and modified (2) active layer of TFC-FO membranes.**

Page 7 Figure S3 Water flux of the pristine and modified TFC-FO membranes determined by the FO-Cell using synthetic wastewater as the feed solution and 1 M NaCl solution as the draw solution.

Page 8 Figure S4 CLSM images of bacteria attached to the surface of the pristine (a) and modified (b) TFC-FO membranes.

Page 9 Table S3 Properties of the foulants collected from the fouled pristine and modified TFC-FO membranes.

Page 10 Figure S5 Integrated CLSM images of polysaccharides (blue), proteins (green) and microorganisms (red) in the biofouling layer of the pristine (a) and modified (b) TFC-FO membranes.

Page 11 Table S4 Biovolume and mean thickness of the pristine and modified TFC-FO membranes.

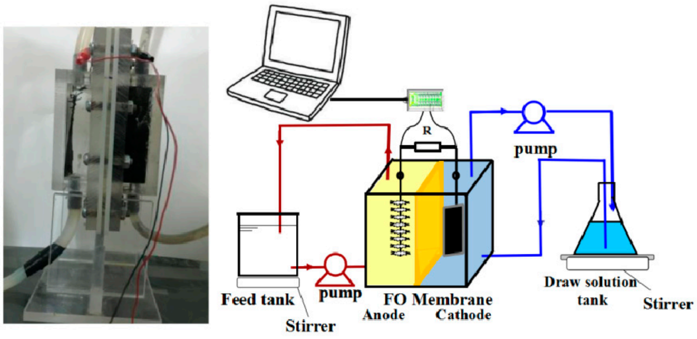

Figure S1 Schematic diagram of the OsMFC.

Table S1 Composition of the synthetic wastewater.

Composition	Concentration (mg/L)
glucose	230.0
sodium acetate anhydrous	40.0
peptone form fish	60.0
beef extract	20.0
NH_4HCO_3	170.0
KH_2PO_4	12.0
$CaCl_2$	1.2
$MgCl_2 \cdot 6H_2O$	2.4
$FeCl_3 \cdot 6H_2O$	1.0
$NaHCO_3$	198

Table S2 EDX analyses of the pristine and modified active layer of TFC-FO membranes.

Element	Wt.%	
	Pristine membrane	Modified membrane
C	79.41	77.76
O	13.20	11.92
S	7.39	8.18
Ag	-	2.14

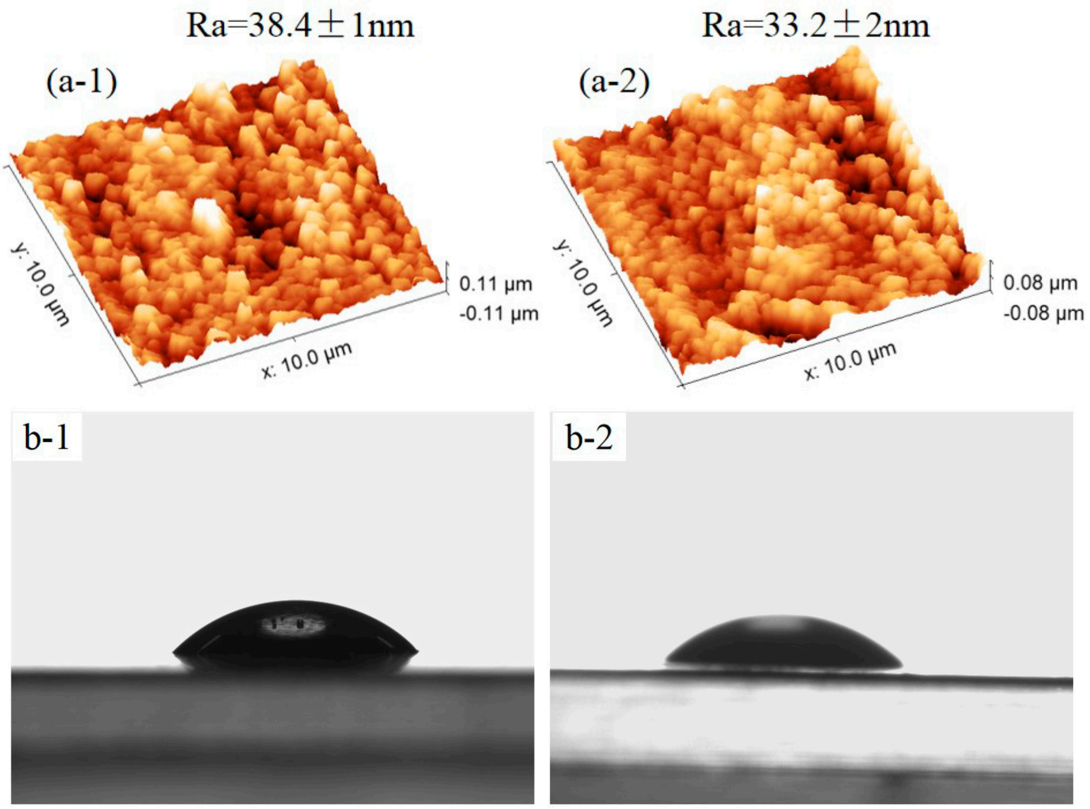

Figure S2 AFM micrographs (a) and contact angle micrographs (b) of the pristine (1) and modified (2) active layer of TFC-FO membranes.

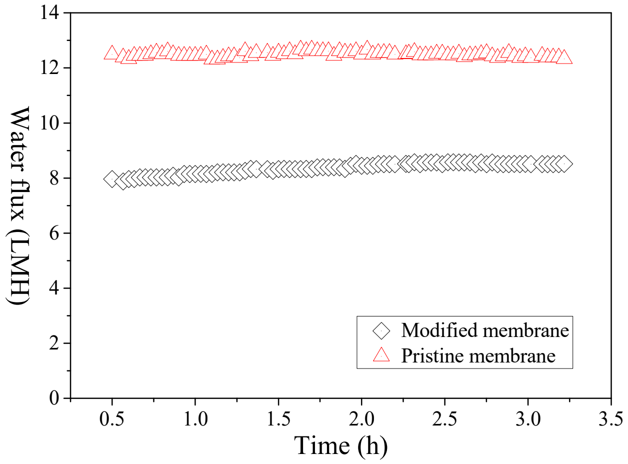

Figure S3 Water flux of the pristine and modified TFC-FO membranes determined by the FO-Cell using synthetic wastewater as the feed solution and 1 M NaCl solution as the draw solution.

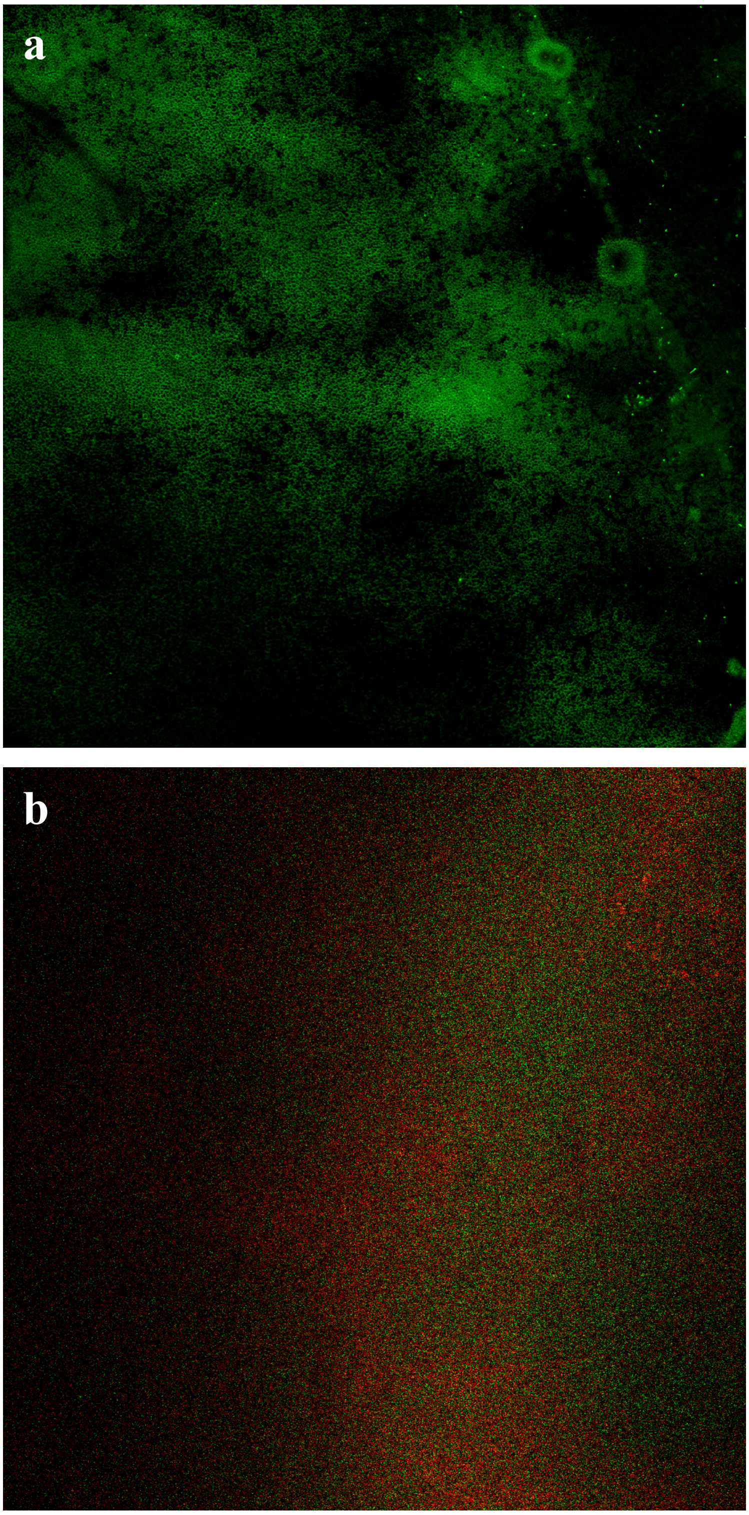

Figure S4 CLSM images of bacteria attached to the surface of the pristine (a) and modified (b) TFC-FO membranes.

Table S3 Properties of the foulants collected from the fouled pristine and modified TFC-FO membranes[a].

Index	Pristine membrane	Modified membrane
MLSS (mg/m^2)	32.0 ± 1.5	27.2 ± 1.2
MLVSS (mg/m^2)	28.8 ± 1.8	24.0 ± 1.1
MLVSS/MLSS	0.90 ± 0.08	0.88 ± 0.07

[a] Values are given as mean values ± standard deviation (number of measurements: n = 3).

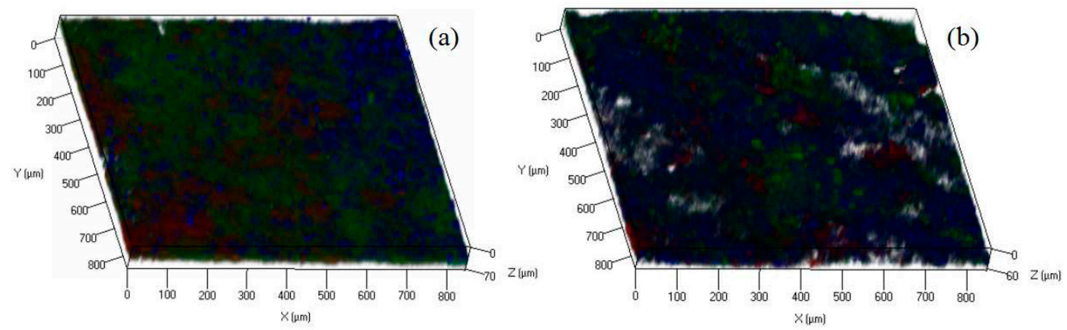

Figure S5 Integrated CLSM images of polysaccharides (blue), proteins (green) and microorganisms (red) in the biofouling layer of the pristine (a) and modified (b) TFC-FO membranes.

Table S4 Biovolume and mean thickness of the pristine and modified TFC-FO membranes[a].

Components	Membrane Type	α-D-Glucopyranose	β-D-Glucopyranose	Proteins	Total cells
Biovolume	Pristine membrane	1.99 ± 1.68	11.53 ± 0.18	20.54 ± 0.10	15.44 ± 6.38
($\mu m^3/\mu m^2$)	Modified membrane	0.03 ± 0.04	11.95 ± 0.26	15.08 ± 0.08	9.67 ± 0.66
Mean thickness	Pristine membrane	28.62 ± 1.78	31.86 ± 0.56	38.31 ± 0.91	36.37 ± 1.41
(mm)	Modified membrane	26.43 ± 2.62	33.98 ± 1.70	36.95 ± 2.57	39.63 ± 0.06

[a] Values are given as mean values ± standard deviation (number of measurements: n = 3).
